# Dr. Myron H. Mills

**Published:** 1897-09

**Authors:** 


Dr. Myron H. Mills, of Mount Morris, one of the oldest and most prominent residents of that village, died at his residence on Saturday, August 14, 1897, of apoplexy. He was a son of William Mills, who built the first house on the Mount Morris tract.

Dr. Mills was well known throughout Western New York. He was president of the Livingston-county Historical Society, of the Livingston-county Pioneer Society and of the Mills Water-works Company of that place. He was the author of much Indian history under the familiar nom de plume of "Cornplanter." He was graduated from the Geneva Medical College in 1844, enlisted as a private in the Mexican war and was promoted to be an assistant surgeon U. S. Army. He is survived by a widow, two daughters and two sisters.

